# Studying policy implementation using a macro, meso and micro frame analysis: the case of the Collaboration for Leadership in Applied Health Research & Care (CLAHRC) programme nationally and in North West London

**DOI:** 10.1186/1478-4505-10-32

**Published:** 2012-10-15

**Authors:** Sarah EM Caldwell, Nicholas Mays

**Affiliations:** 1 Department of Health Services Research and Policy, Faculty of Public Health and Policy, London School of Hygiene and Tropical Medicine, 15-17 Tavistock Place, London, WC1H 9SH, UK; 2 NIHR CLAHRC for Northwest London, 4th Floor, Lift Bank D, Imperial College London, Chelsea & Westminster Hospital NHS Foundation Trust, 369 Fulham Road, London, SW10 9NH, UK

**Keywords:** Policy implementation, Policy analysis, Frame analysis, Knowledge translation, Health services research, Translational gap

## Abstract

**Background:**

The publication of *Best research for best health* in 2006 and the “ring-fencing” of health research funding in England marked the start of a period of change for health research governance and the structure of research funding in England. One response to bridging the ‘second translational gap’ between research knowledge and clinical practice was the establishment of nine Collaborations for Leadership in Applied Health Research and Care (CLAHRCs). The goal of this paper is to assess how national-level understanding of the aims and objectives of the CLAHRCs translated into local implementation and practice in North West London.

**Methods:**

This study uses a variation of Goffman’s frame analysis to trace the development of the initial national CLAHRC policy to its implementation at three levels. Data collection and analysis were qualitative through interviews, document analysis and embedded research.

**Results:**

Analysis at the macro (national policy), meso (national programme) and micro (North West London) levels shows a significant common understanding of the aims and objectives of the policy and programme. Local level implementation in North West London was also consistent with these.

**Conclusions:**

The macro-meso-micro frame analysis is a useful way of studying the transition of a policy from high-level idea to programme in action. It could be used to identify differences at a local (micro) level in the implementation of multi-site programmes that would help understand differences in programme effectiveness.

## Background

In October 2008, the National Institute for Health Research established nine Collaborations for Leadership in Applied Health Research and Care (CLAHRCs) across England with five years of funding and a mission to ‘bring together universities and their surrounding NHS organisations (including primary care) to test new treatments and new ways of working’ [1]. Whilst there is some information on the origins of the CLAHRCs, the national-level policy and the programme’s aims and objectives have not been systematically analysed. This lack of a single account is significant given the interest in the programme as a “natural experiment” in different modes and methods of translating knowledge to practice in that each CLAHRC proposed and has implemented a different approach to knowledge translation. In order to assess whether or not the nine CLAHRCs have fulfilled the aims and objectives of the national programme, it is important to have a sense of whether the understanding of individual CLAHRCs matched the intentions of the Department of Health and the NIHR. What follows is an analysis of the degree to which there was, or was not, a shared understanding of the aims and objectives of the CLAHRC programme starting at the strategic health system level (the Department of Health and the NIHR) and then narrowing to a local health economy (the North West London CLAHRC).

The publication of *Best research for best health*[2] and the “ring-fencing” of health research funding marked the start of a period of change for health research governance and the structure of research funding in England. Under the direction of the Department of Health’s Director-General of Research and Development, the English National Institute for Health Research (NIHR) was founded with a mandate to establish programmes that work across sectors (public, academia, charitable, industry), and to ‘maintain a health research system in which the NHS [can support] outstanding individuals, working in world class facilities, conducting leading edge research focused on the needs of patients and the public’ [1,3]. Research commissioned through the NIHR focuses primarily on providing the evidence needed to support improved quality of care, patient outcomes and investment decisions – in essence, applied research that ‘[plays] a crucial role in bridging the gap in translating research from invention to diffusion’ [1]. The CLAHRC programme is one important part of the NIHR’s infrastructure for such knowledge translation.

Since their inception, the CLAHRCs have been held aloft by the Department of Health and the NIHR as a novel way of funding and organizing research to directly address the “second gap in translation,” identified by Sir John Cooksey in *A review of UK health research funding*[4,5]. If the “first gap in translation” refers to the difficulty of harnessing the ideas of basic scientists and clinician-scientists, then the “second gap in translation” relates to the way in which new knowledge and processes diffuse, or fail to, across the health system [[4]: 86]. The NIHR’s briefing document on the CLAHRCs defines the task of filling the “second gap in translation” as ‘the evaluation of those new interventions that are effective and appropriate for everyday use in the NHS and the process of implementation into routine clinical practice’ [5]. It also identifies the programme as a response not only to Cooksey [5] but also to the call by the English Chief Medical Officer’s High Level Group on Clinical Effectiveness to ‘harness better the capacity of higher education to support initiatives to enhance the effectiveness and efficiency of clinical care’ [6]. This paper deals specifically with the implementation of the CLAHRC policy as a programmatic means to address the ‘second translational gap’, by tracing the understandings of the aim and objectives of the programme between the national level and the local level in North West London. The analysis does not attempt to evaluate how well the structure, management and approach of North West London or any of the other eight CLAHRCS have narrowed the ‘second translational gap’ at the local level. By contrast, the NIHR Service Delivery and Organisation (SDO) programme has a research programme to tackle this. To the authors’ knowledge the primary focus of these studies is not the implementation of the CLAHRCs as a policy and programme but instead on the effectiveness of the CLAHRCs (and the CLAHRC model) in closing the second translational gap.

### Conceptual approach

The intention is to look not only at the mechanisms by which the CLAHRCs moved from policy idea to programme implementation, but to analyse how the participants at each level framed their understanding of the policy and programme, and to determine the degree of congruence within and amongst the levels in their understandings of the programme. Using a variant of Goffman’s frame analysis [7], as adapted by Pope et al. [8], this paper investigates the conceptualization of the CLAHRC initiative in the Department of Health as a policy (at the macro level), then as a programme as it was shaped through the funding and governance structures of the NIHR (at the meso level) and, finally, as it was implemented in North West London (at the micro level). The premise behind the macro/ meso/ micro-level framing is the idea that ‘to understand the pace, direction and impact of organizational innovation and change we need to study the interconnections between meanings across different organizational levels’ [[8]: 59]. In this case, the different organisational levels are within the health research and public health care delivery (NHS) systems in England.

Goffman identified a “frame” – or framework – as “schemata of interpretation” to locate, perceive, identify and label situations, experience, meaning, etc. [7]. Over time other scholars have expanded this definition to include additional factors such as resource utilization and politics to understand character, causation and the course of change [9]. By analysing understanding and action at multiple levels more of the influences on development and interpretation of policy become evident. The macro, meso and micro levels of data collection and analysis help categorize the actors and illuminate how the idea of the CLAHRC, both as a national policy and a local programme, has been generated, diffused, shaped and changed [[9]: 612]. Organisational theorists distinguish the image (the way ‘organisational elites would like outsiders to see the organization’) and identity (‘an organisation’s members’ collective understanding’) of an organisation [[10]: 64–65]. In the context of this study, image and identity are represented by how CLAHRC's mission, mandate and objectives are defined and understood at the local (micro) level. The inclusion of an analysis of how the CLAHRC team in North West London sees itself is important because it frames how the micro level (here the CLAHRC in North West London) understands itself in relation to the national level policy and meso level programme.

To aid the analysis and discussion of the results it is useful to set out a working definition for each frame/ level. The definitions are consistent with the theoretical model, but are specific to this case study.

#### Macro-level frame

The orientation of the macro frame is the policy context that established the “second translational gap” as something requiring government action. At this level, the CLAHRC programme existed largely conceptually. The actors working in this frame were the visionaries behind a policy and funding scheme that would specifically address the second gap in translation in the context of the larger national health research infrastructure, and in relation to other health research funding initiatives, such as the Academic Health Science Centres, the Health Innovation and Education Clusters, and the Biomedical Research Centres and Units.

#### Meso-level frame

The meso frame is where policy begins to take shape as a specific programme. Here the CLAHRCs began to take shape as more than a high level concept, and it was at this level that the policy was negotiated into a programme with specific scope and deliverables. The transformative nature of this frame means that there was the greatest potential for misunderstanding or misinterpretation of aim and objectives at this level.

#### Micro-level frame

In practice, there are nine micro frames, one for each CLAHRC. This paper focuses solely on the North West London CLAHRC’s understanding of the macro and meso levels and its understanding of itself as an organisation in its local context, including its ability to fulfil the proposed CLAHRC model as policy and programme (the micro level frame). The conclusions drawn at this level are not presumed to be directly applicable to the other eight CLAHRCs.

## Methods

The data for this study were collected concurrently as part of one of the author’s (SC’s) doctoral programme. SC worked in the North West London CLARHC on primary research and project evaluation, and used the opportunity to conduct embedded observational research. Staff and other CLAHRC collaborators were informed of this dual role. The four months with the CLAHRC was used to learn more about the methods and approaches the North West London employs, understand the CLAHRC policy and programme from the perspective of those working there, and to inform and aid the selection and collection of documents. This working relationship also facilitated access to interview data collected by the Imperial College Business School as part of their prospective evaluation of the North West London CLAHRC. The opportunity to be able to add embedded research to the data collection was valuable in the data analysis phase. In particular, being party to the day-to-day conversations of the CLAHRC and listening to how their projects were designed to fulfil their mission and objectives helped to understand the North West London CLAHRC’s perception of the national CLAHRC policy and programme, and how it was responding to them.

The documents selected for inclusion in the analysis were based on: both authors’ knowledge of the CLAHRC programme; key informant recommendations; follow-up of references contained within other documents; and, a request for information from the Department of Health under the Freedom of Information Act. Each document was skimmed for relevance and then read in detail after being deemed to be of potential significance. A table was created to extract and code the data under thematic headings. Following the review of all documents the extracted data was re-reviewed until no new headings emerged. In the end, the complete list of headings included: aim; objective; values; goals; vision; method; purpose; scope; step-change; partnership; team; drivers; funding; and, evaluation. A column for comments and observations from the embedded research was included alongside the data listed in each thematic code. This same method of summarising and coding was also used for the interview data discussed below.

Interview data were collected or obtained for analysis at the micro, meso and macro levels, representing a total data set of 21 interviews. SC conducted the interviews with informants at the macro and meso levels and, with permission, used the Imperial College Business School data for the micro level. At the macro and meso levels, informants were selected based on their involvement in the development or delivery of the CLAHRC programme at a national level. Names and positions of interest were established from the document analysis and through conversations with others knowledgeable about the programme and/ or the policy context from which it emerged. At the macro level interviewees (n=4) had a largely strategic role in the conceptualisation of the CLAHRCs as a policy, whilst at the meso (n=3) level informants were more closely involved in management and delivery aspects of the CLAHRCs as a programme at the national level. Most of the interviews (n=6) were conducted face-to-face with one by telephone. As the sample is small, though comprehensive for the scope of the project, it is difficult to provide further information about the informants without the risk of disclosing their identity; instead, they are simply referred to by number.

To establish the nature of their involvement in the CLAHRC programme, interviewees were first asked about their relationship to the programme. Following this, a semi-structured interview was conducted using open-ended questions to elicit the informant’s understanding of the aims and objectives of the CLAHRC programme, the policy context as they understood it and whether their understanding had changed over time. They were also asked to provide examples of work that any of the nine CLAHRCs were undertaking that, to them, represented the vision for the programme. The last question in each interview was an opportunity for the respondent to add anything to the interview that they felt was significant, interesting or important. This was often the most informative question, and in many cases led to one or more additional avenues being explored.

Given the multiple, overlapping, ongoing evaluations of the CLAHRC programme locally and nationally, one of the authors (SC) was granted full access to anonymized interview transcripts for the micro level analysis to alleviate fears of interview fatigue. A first round of interviews with the North West London CLAHRC staff (n=14) had been conducted by the Imperial College Business School in spring 2009 and one of the questions asked was: “how would you describe the CLAHRC?” which was viewed as a proxy for how staff understood the mission and objectives of the policy and programme. In exchange for access to these data, SC did not re-interview staff to question them specifically about their understanding of the aims, objectives and origins of the CLAHRC programme.

The use of interviews from two different time periods and conducted by different interviewers for slightly different purposes is an acknowledged limitation of this study. However, interview data were supplemented and corroborated by SC from her period of embedded research with field notes, informal conversations with staff, and attendance at weekly team meetings and other staff and stakeholder events. Also, the fact that these interviews had been undertaken by researchers outside the CLAHRC may have been stronger methodologically than SC interviewing colleagues in the CLAHRC with whom she was working at the time.

## Results and discussion

This section presents the findings from the documentary analysis and interviews in two different ways – for each of the individual levels and across frame boundaries. The focus is on establishing the context for action and understanding in each frame, and then elaborating how understanding of the CLAHRC programme does, or does not, change across and between levels (see Additional file 1: Table S1).

### Macro frame

The basis of the policy to address the “second translational gap” was the release of three official reports. *Best research for best health* set out five goals for five years that centred on strengthening the place of research in the English NHS [[2]: 2]. The strategy, as commented on by Hanney et al., ‘sensibly both builds on recent progress and tackles acknowledged weaknesses . . . through a system that should improve and simultaneously expand translational, clinical and applied health research, and increase the extent to which research is then used in the health care system’ [[11]: 28]. Implicit throughout *Best research for best health* is the need for the government to do a better job of funding the full spectrum of health research, and in particular research that addresses “translational gaps” [2,11]. This can be recast as the need to implement policies and programmes targeted at tackling translational gaps.

The second significant policy driver - the Cooksey Review – similarly recommended a change in the way that health research was commissioned in order to reach a ‘position where research and innovation are “hardwired” into the NHS as a core objective alongside service provision and teaching’ [[4]: 67]. In particular, funding arrangements should be designed more comprehensively and coherently to support the translation of ideas into practice [4]. The language that would come to define and describe the CLAHRC programme has its origins in the Cooksey Review and the Implementation Plans for *Best Research for Best Health*. As one informant noted:

it was very clear the, what, what they were trying to do was address the second Cooksey gap, the translational gap . . . [s]o, in that sense this competition was congruent with research understandings about how knowledge moves about . . . it was congruent with contemporary thinking on knowledge mobilization. [P006]

The third major driver was the final report of the High Level Group on Clinical Effectiveness chaired by Sir John Tooke. Though commissioned by the Chief Medical Officer (CMO) in England to look specifically at clinical effectiveness, Recommendation 4 has since become a general guiding tenet in English translational research action: ‘we recommend that the Health Service harnesses better the capacity of higher education to assist with this agenda through promoting the development of new models of community-wide “academic health centres” to encourage relevant research, engagement and population focus and embed a critical culture that is more receptive to change’ [[6]:14]. By encouraging a new relationship between the NHS and higher education, the belief was that each would leverage the knowledge and skills of the other to make research and joint work permanent features of the health care culture [6].

It was in specific response to this recommendation that the CLAHRCs were first proposed to narrow the “second translational gap”. In his forward to the Tooke report, the CMO wrote: ‘I am very pleased that at the same time as this report is being published, the Department of Health’s Research and Development Directorate is announcing the establishment of NIHR Academic Health Science Centres of the Future. These will develop innovative models for conducting applied health research and translating research findings into improved outcomes for patients, through partnerships between academia and the NHS across the health community covered by the Centre’ [6]. The Academic Health Science Centres of the Future would soon be renamed the CLAHRCs.

These three drivers came together to create what Kingdon [12] calls a “policy window” or opportunity for change. External public pressure (Cooksey and Tooke) to respond to a significant issue occurred at the same time as internal recognition (*Best Research for Best Health)* of the need to fill a gap in a particular area of health research funding, all within the context of a reorganization of health research governance that provided the opportunity, structure and protected resources to make the change happen [P001]. However, there was not complete unity within the Department of Health as to how to respond to the call to close the second gap in translation, for example, some policies and programmes, such as the Health Innovation Education Clusters (HIECs) were developed and launched at nearly the same time as the CLAHRCs, but under a separate process. Key informants felt that:

although there’s been a lot of rhetoric about the second gap in translation and research about the reasons for it, there was no consensus view of, of what needed to be done and who the key players were and what the key levers were. [P004]

there is a degree of policy competition within the Department of Health, in the sense that a number of initiatives that on the face of it seem to be doing similar sorts of things . . . in a sense, [they] are all doing related things in rather different ways but they are articulated by different parts of the policy machine. [P006]

Lord Darzi’s NHS White Paper *High quality care for all*[13] is often identified as one of the policy drivers of the CLAHRCs, but the timing of its release meant that it could not have directly influenced the thinking about the policy’s aim or objectives. Rather, the future state of the NHS that the White Paper envisages coincided with the CLAHRC bidding process and the finalisation of details about how individual CLAHRCs would implement their vision for closing the “second translational gap”. For this reason, the Darzi report should be considered more as an indirect influence on the subsequent meso and micro levels, rather than a high-level driver of the macro frame.

### Meso frame

The importance of the meso frame lies in how the actors working at this level translated their understanding of the aims and objectives of the CLAHRCs as a policy into a working structure for an implementable programme. Several informants within this frame felt that there had been specific models, or the work of prominent academics in mind, and that these had informed the crafting of the aims and objectives in the macro frame [P002].

[the CLAHRC competition] was congruent with research understandings about how knowledge moves about and how, and where, it gets stuck. [P006]

In contrast, interviewees from the macro level did not attribute the vision for the CLAHRCs to any particular body of scholarship or pre-determined knowledge translation models [P001, P004], choosing instead a policy science narrative about how different sources of evidence and knowledge come together to inform decision-making [14].

there’s been a lot of rhetoric about the second gap in translation and research about the reasons for it, there was no consensus view of, of what needed to be done and who the key players were and what the key levers were in, to achieving, whatever recognized needed to happen. So, we were deliberately not prescriptive and given the needs of different communities and the infrastructure that’s available in different communities I think what the CLAHRC programme will show is that you don’t need to impose a one-size fits all solution to, to achieving things. [P004]

Despite this difference of views there was a shared belief that the CLAHRCs were something new – both in terms of structure and function. In the earliest announcements, the term “pilot” was included, but always used loosely, and was later dropped with no public acknowledgment of the change or what it denoted. Whilst several of those interviewed mentioned the change, none could offer an explanation as to why, suggesting instead that the concept of “natural experiment” was more applicable both in terms of structure and functioning.

I was suggesting that they used to be called pilots, the pilot word is gone now . . . so that’s worth just noting that . . . uh, yeah, I think it’s no longer seen as a pilot. [P002]

. . . they have been self-forming, their compositions are different, their funding partners are very different and provided that we’re convinced that they are focusing on, on the end-game, um, then we are, you know, keen to, sort of, let them get on with it and then at the end, if you like, at the end of the experiment we then look at what’s happened. [P004]

This switch from pilot to natural experiment is notable because a “pilot study” connotes testing a particular idea or design before scaling-up, whilst a “natural experiment” is less controlled and without a clearly defined or prescribed structure that must be adhered to. The flexibility of the “natural experiment” designation was fully embraced by the NIHR as it drafted the Call for Proposals and other guidance documents to turn CLAHRC policy into a programme, and is evident in the nine different approaches that the nine CLAHRCs have taken towards implementing their understanding of how to address the “second translational gap”.

they were all doing things slightly different things and they’d all organized themselves in slightly different ways so that natural experiment provided a great opportunity to learn about how partnerships between research producers and research users function. [P006]

Awareness of the potential for misunderstanding about how the CLAHRCs were to differ from other NIHR-funded research schemes led to a short but active period of negotiation between the macro and meso levels, so that there were agreed common understandings of the policy’s purpose and its parameters as a programme [P002]. For example, it was decided that the CLAHRCs were to differ from other NIHR-funded research in terms of: the temporal proximity between research and practice; funding that could be used to directly support implementation; and, being based on a partnership between an academic institution and a ‘consortium of NHS organisations’ in a local “health economy” rather than a single NHS partner [[15]: 1–2].

However, the number of questions pertaining to definitions of “applied health research”, “implementation” and various aspects of partnership included in an issue note published by the NIHR following a briefing with potential applicants [15] indicates the challenges the respondents had in understanding how the CLAHRCs could uniquely respond to the “second translational gap” and be distinct from other NIHR funding schemes aimed at translational gaps. For example, the quick renaming of the CLAHRCs (from Academic Health Science Centres of the Future) was ‘to ensure that appropriate emphasis was given to the collaborative nature of these partnerships and their role in both applied health research and implementation of research evidence, and to avoid any confusion with Academic Health Science Centres which are quite different in purpose and structure’ [[15]: 1].

The main finding emerging from the analysis of understandings in the macro and meso frames is that as national policy became a specific programme, the focus was consistently on finding new ways of addressing the “second translational gap”. Though not all informants agreed as to whether the CLAHRCs were a variation on other knowledge transfer/ exchange models or something novel, there was broad agreement that the CLAHRCs had the potential to expand both the academic and policy fields’ understanding of the connection between knowledge production and its application.

### Micro frame

At the micro level those working in this frame must translate their understanding of how policy is expressed as a programme (framework) into day-to-day work, a process shaped by organisational structure and shared narratives. The perception of the members of the North West London CLAHRC of how the programme was framed at the macro and meso levels shaped their understanding of what they were meant to be implementing.

An analysis of several early documents produced by the North West London CLAHRC shows that there was a nuanced understanding of what the CLAHRC was meant to do locally and as part of the national programme [16,17]. The North West London bid document states that the CLAHRC would ‘[streamline] patient care, [reduce] variation and [improve] equity of care, supporting care in the community and reducing hospital admissions’ [18], whilst the press release from Imperial College describes the CLAHRC as a ‘Collaboration [that] will develop service innovations to improve the care of acutely ill patients and patients with chronic disease across different NHS organisations’ [19]. These descriptions show that micro-level understanding of the CLAHRC was oriented towards tangibles such as the methods for closing the “second translational gap”, but that there was also an understanding of where the North West London CLAHRC fit into a wider system. In response to interview questions, respondents had a generally good grasp of the origins of the CLAHRCs in the Cooksey and Tooke recommendations (macro level), and their freedom to experiment with new and different organisational structures (meso level). However, there was less clarity about how this general level of understanding was intended to be implemented at an operational level:

we’ve been struggling with this [laughter], um, I usually describe it as an attempt to put… to apply the findings from academic health research, um, to make them more applicable to health services, you know? Um, to draw closer links between an academic approach to health research and what it is that NHS services actually need, because I think there’s a sort of, a mismatch between them. [PRO-I 006]

the aims and objectives are taken from what CLAHRC, overall national CLAHRC objectives are to, you know, to get research in the practice, to build researching capacity, etc., etc. But the… our objectives in terms of trying to build this into a robust evaluation framework with iterative feedbacks within a learning organization. [PRO-I 012]

The policy and programming work at the macro and meso levels established a mandate for the CLAHRCs, but work and framing at the micro level was then required to create a distinctive and complementary organisational mission and related objectives. In the North West London CLAHRC there is evidence of some tension between the macro/ meso and the micro levels as far back as the North West London bid. The principal investigators proposed to make testing and implementing quality improvement methods the centre piece for addressing the “second translational gap” [18], rather than choosing specific areas of chronic disease or public health interventions to work on, as had been the national programme’s original intention [20]. However, in the letter notifying the North West London CLAHRC that it had been successful in the competition, the assessment Panel noted that the proposed approach was compelling and congruent with the goals of the funding scheme. This provides a glimpse into what at the macro and meso levels was considered to be an acceptable micro-level interpretation of the programme, and indicates that the mission that the North West London CLAHRC envisaged for itself was acceptable in terms of the vision of the national CLAHRC programme.

It should be born in mind that some of the reconciliation of macro-level aims and objectives with micro-level understanding was related to the hiccoughs of starting anything new. Observations of the North West London CLAHRC are, not surprisingly, that tensions were more pronounced at the staff level than among its leaders. Those who initiated the bid had a particular vision for how to adapt and adopt quality improvement methods to fulfil the macro and meso-level objective of closing the second translational gap. This means that while the leaders of the North West London CLAHRC shared an understanding with their counter-parts at the macro and meso-level, those at the micro level were still developing the confidence that they were doing what they were supposed to be doing.

I really struggled, absolutely struggled, which I don’t think I’m the only one, to kind of get a grip of what CLAHRC is really trying to achieve. It is really different. [PRO-I 005]

I’m, kind of, looking back at the bid documents, and I think feeling some frustration, because they’re all very vague. It’s all very high level and fancy words, and I’m, kind of, like yes, but that doesn’t tell me how to do anything. [PRO-I 007]

### Common understanding or implementation gap?

Whilst the CLAHRCs continue to evolve as means for funding and undertaking and implementing translational research, through a national programme and as nine individual organizations with nuances in their understanding of the aims and objectives at each level, in reality, they have a great deal in common [7]. The aims and objectives laid out in the original policy document, and held in the heads of its crafters, seem to be present in the spirit and purpose behind the work at the micro level in North West London. Interviews with those whose primary role was located in either the macro or meso frame revealed that they did not feel as if their understanding about the CLAHRCs’ aims and objectives had changed (“no, my view of it didn’t change” [P006]). And though many of those working at the meso/ micro levels used metaphors related to journeys and travel, the differences between those at the different levels are more reflective of people’s roles in the process (policy or programme), than fundamentally different understandings; a proposition that aligns with Goffman’s frame theory [7].

I think that everybody’s understanding has unfolded . . . I think we’ve all learnt over the last year, you know, what, what we’re trying to do and how, and how we’re trying to do it in a bit more of a clear cut way. So, yeah, it’s just become, I mean the CLAHRC is an evolving organization, it’s changing all the time, um the overall objectives might be the same but the means of delivering them are clearly evolving and getting more refined as time goes by. [P005]

the journey so far has made that clearer to people, and with that clarity the excitement comes. [PRO-I 002]

I think my understanding of CLAHRC will always evolve . . .my view of it has definitely evolved. I think I would imagine I have perhaps seen myself, looking through other people who fully understand CLAHRC as it is to us, and I think what’s very interesting now is that it comes CLAHRC to lots of other people. [PRO-I 010]

The government’s official description of the programme has a practical tone, stating, for example, that ‘the CLAHRCs and their evaluation will help us to understand how best to get good research evidence into practice,’ [[21]: 28] but, if anything, macro-level actors are even more positive, both in terms of what they say about what has already been achieved and what might be accomplished by the end of the five-year funding term.

it’s all fairly abstract. I mean . . . it was fascinating to see them in practice, it’s actually a very interesting scheme. [P002]

CLAHRC is unique in the fact that it works across communities. [P003]

I think it’s one of the most exciting things to come out of NIHR . . . it really does try to um break-out from the twin communities model of knowledge transfer that we’ve got bogged down in . . . I’ll be very interested to see how it works. But of course it’s, it’s a real challenge to make work in practice. [P006]

In interviewing those who were involved in the development of the policy and programme aims and objectives, there was a sense that the CLAHRCs had generally already surpassed expectations, and that, in different ways, the micro level understanding of what they were meant to be doing and achieving was aligned with what was framed in the initial policy and programme. This is not to say that there was no skepticism about the initiative (‘I think it’s all very wooly, to be honest’ [P005]), but this applied to the minority.

## Conclusion

The purpose of this research was to study policy implementation; more specifically, it was to assess the extent to which understanding of the national aims and objectives of the CLAHRCs was translated from national policy (macro level) to a programme (meso level) and, in turn, implemented locally in North West London (micro level). Across the macro and meso levels there are strong indications of a shared understanding. In effect, the programme as implemented matches the policy as envisioned. Further, at least in North West London, local level actors understand the aims and objectives of the policy and programme in a very similar way to the macro and meso level understandings, In this specific sense, implementation of the CLAHRC policy and programme can be seen to be a “success”. What makes this result noteworthy is the lack divergence between understandings at the three levels. While a strong degree of similarity at the strategic and programmatic levels might be expected, its extension to the local (micro) level was less assured.

The implication for those evaluating the effectiveness of the CLAHRC programme and the nine CLAHRCs individually, is that it seems that they have indeed been pursuing similar goals, and that any differences in achievements and outcomes are likely to be attributable to either the context in which they are working, or their theoretical approach to knowledge translation. This should greatly simplify the evaluation task. As the CLAHRCs were envisioned as a means to close the second translational gap, it is heartening that, at the macro and meso levels, the programme itself did not fall victim to a breakdown about aims and objectives between the articulation of the policy idea and the development of a programme with a proposed structure. The process by which the CLAHRCs moved from policy to programme is then perhaps an example for policy makers of how successfully to move a concept into practice. Whilst new ideas will not always flourish, the negotiation between the macro and meso levels about what the policy would look like (for example, in how it was described in the Call for Proposals), and then the cooperative work with potential bidders to build understanding, appears to have paid dividends in relation to the CLAHRCs. Where all the parties involved are working with a common understanding of purpose and objectives, there is a greater likelihood of success.

The broader implication of the analysis for the study of policy implementation relates to the usefulness of Goffman’s frame analysis. One of the difficulties in studying the policy process is discerning the motivations of those involved, how ideas become decisions and how decisions become programmes. Studying the actors and the steps in the process using the notion of policy “fames” at the macro, meso, and micro levels allows for each level to be contextualized and studied in its own right, and then linked together to examine how translation of policy ideas does or does not occur between levels. Working in and across these three levels can help with understanding the success of a policy’s implementation, or to pinpoint where and why it broke down. This last point is particularly germane to policies and programmes with multiple sites, like the CLAHRCs, where it may be important to be able to differentiate the outcomes at a local level in each site, from those of the overall programme. Therefore, this research contributes to both the policy process and knowledge translation literatures on how a shared understanding of aims and objectives affects the successful transformation of a policy into an actionable programme. This knowledge, along with the utility of frame analysis for studying implementation, can be used in developing future initiatives similar to the CLAHRCs in either form or function.

## Abbreviations

CLAHRC: Collaboration for Leadership in Applied Health Research and Care; NIHR: National Institute for Health Research; NHS: National Health Service.

## Competing interests

Neither author has any competing interests to declare.

## Authors' contributions

SC conducted the primary research including data collection and document analysis. NM and SC worked jointly on analysis and discussion of findings and conclusions. SC prepared the first draft of the entire paper. NM reviewed several versions of the paper and provided constructive, substantive comments and suggested changes. NM is SC’s doctoral supervisor. All authors read and approved the final manuscript.

## Supplementary Material

Additional file 1 **Table S1.** Box: Three frames, three levels of understanding and types of work. Click here for file
