# Supplementary material for: Studying policy implementation using a macro, meso and micro frame analysis: the case of the Collaboration for Leadership in Applied Health Research & Care (CLAHRC) programme nationally and in North West London
Source: Health Res Policy Syst. 2012 Oct 15;10:32. doi: 10.1186/1478-4505-10-32 (PMC3503608; doi:10.1186/1478-4505-10-32)
Supplement: Supplementary file 1 [file 1478-4505-10-32-S1.docx]

Box 1 Three frames, three levels of understanding and types of work

Macro		Government, strategic frame

Meso			Funding, programmatic frame

Micro			Implementation, local-level frame
